# Insights Into How Digital Health Interventions Shape Outcomes for Emerging Adults Living With Type 1 Diabetes: Qualitative Realist Process Evaluation

**DOI:** 10.2196/70401

**Published:** 2025-09-05

**Authors:** Ruoxi Wang, Balpreet Panesar, Mikayla Sonnenberg, Alanna Landry, Anne-Sophie Brazeau, Marley Greenberg, Meranda Nakhla, Ian Zenlea, Elise Mok, Jessica C Kichler, Ellen B Goldbloom, Gillian L Booth, Mélanie Henderson, Rayzel Shulman, Laura Desveaux

**Affiliations:** 1 Institute for Better Health Trillium Health Partners Mississauga, ON Canada; 2 Division of Endocrinology The Hospital for Sick Children Toronto, ON Canada; 3 Child Health Evaluative Sciences SickKids Research Institute Toronto, ON Canada; 4 CDE Oak Valley Health Markham, ON Canada; 5 School of Human Nutrition McGill University Montreal, QC Canada; 6 Diabetes Action Canada Canadian Institutes for Health Research (CIHR) Strategy for Patient-Oriented Research Network in Chronic Disease Toronto General Hospital Toronto, ON Canada; 7 McGill University Montreal Children’s Hospital Montreal, QC Canada; 8 Faculty of Medicine and Health Sciences McGill University Montreal, QC Canada; 9 Research Institute of the McGill University Health Centre Montreal, QC Canada; 10 Temerty Faculty of Medicine University of Toronto Toronto, ON Canada; 11 Institute of Health Policy, Management and Evaluation University of Toronto Toronto, ON Canada; 12 Centre for Outcomes Research and Evaluation Research Institute of the McGill University Health Centre Montreal, QC Canada; 13 Department of Psychology University of Windsor Windsor, ON Canada; 14 Children’s Hospital of Eastern Ontario Ottawa, ON Canada; 15 Children's Hospital of Eastern Ontario Research Institute Ottawa, ON Canada; 16 Faculty of Medicine University of Ottawa Ottawa, ON Canada; 17 MAP Centre for Urban Health Solutions Unity Health Toronto Toronto, ON Canada; 18 Department of Pediatrics Université de Montréal Montreal, QC Canada; 19 Centre de Recherche CHU Sainte-Justine Montreal, QC Canada; 20 Department of Social and Preventive Medicine School of Public Health Université de Montréal Montreal, QC Canada; 21 Child Health Evaluative Sciences Sickkids Research Institute Toronto, ON Canada

**Keywords:** digital health, type 1 diabetes, emerging adults, transition care, realist evaluation

## Abstract

**Background:**

Emerging adults living with type 1 diabetes (T1D) need targeted support to equip them with the knowledge and motivation required for self-management, particularly as they transition from pediatric to adult care. While multicomponent digital health interventions have shown promise in addressing their multifaceted needs, traditional effectiveness studies provide little, if any, insights into which components work effectively, how they function, and for whom.

**Objective:**

This study aims to explore the implementation of a multicomponent, text message–based digital intervention (Keeping in Touch; KiT) to provide early insights into which components may shape participants’ transition experiences and how. The secondary objective was to explore which subgroups, defined by individual characteristics, may benefit most from the intervention.

**Methods:**

Embedded within a broader randomized controlled trial, we conducted a qualitative realist evaluation with intervention-arm participants who had engaged with KiT for a minimum of 3 months. One-on-one semistructured realist interviews were conducted in a teacher-learner cycle to test the initial program theory. The initial program theory included several pathways through which the 5 intervention components (ie, T1D self-management information and suggestions, transition support information, problem-solving support, stress management strategies, and transition reminders) were hypothesized to influence a range of theorized outcomes.

**Results:**

A total of 16 interviews were completed with intervention participants. All 5 KiT intervention components were reported to shape participants’ transition experiences positively but to varying degrees. T1D self-management information and suggestions presented a universal positive impact across all participants. However, the effectiveness of problem-solving support and stress management strategies varied depending on participants’ individual characteristics (eg, duration of diabetes, perceived access to information, and baseline diabetes distress). Rather than acting through parallel independent mechanisms, KiT appeared to support participants’ transition experiences via multiple chains of interconnected mechanisms, often beginning with knowledge or reinforcement and contributing to changes in motivation (eg, self-efficacy and diabetes distress). Interview participants described tangible improvement in mechanisms and proximal outcomes (eg, diabetes knowledge and self-efficacy).

**Conclusions:**

A multicomponent, text message–based digital intervention could support emerging adults living with T1D during their transition to adult care by enhancing their knowledge and motivation for self-management. Participant subgroups responded differently to various intervention components, which highlights that one-size-fits-all approaches are likely inadequate. Digital interventions should be developed and studied in a variety of subgroups and contexts to optimize their reach. Interventions for emerging adults living with T1D might benefit from targeting those who are more recently diagnosed with relatively lower baseline levels of diabetes knowledge and self-efficacy or higher levels of diabetes distress.

**International Registered Report Identifier (IRRID):**

RR2-10.2196/46115

## Introduction

### Background

Type 1 diabetes (T1D) often emerges during childhood or adolescence, affecting 1.5 million individuals aged <20 years, with an expected increasing prevalence over the next 20 years [1]. T1D requires lifelong, routine self-management activities and medical follow-ups to prevent acute and chronic diabetes complications and ensure stable health [2]. Emerging adulthood, a period of development spanning approximately from ages 18 to 28 years [3], is a particularly challenging period for those living with T1D not only because of a shifting dynamic in the responsibility for performing daily T1D self-management activities but also because it coincides with major life changes that introduce competing social, academic, and financial needs [4]. One of the common and observable risks introduced during this period is the transition from the pediatric to adult care system [5,6], which has been associated with care gaps that lead to adverse health outcomes and increased acute health care use [7]. There is a practical need for targeted interventions to support emerging adults living with T1D, equipping them with the knowledge and motivation to meet their T1D self-management needs alongside multifaceted and evolving psychological, educational, and vocational needs [7-9].

Increasing attention has been paid to the potential of digital health interventions in offering scalable support for people with T1D. Digital health interventions are widely accessible [10] and can incorporate multiple functions to meet the multifaceted needs of users [11]. Underpinned by a combination of different components, such as educational information [12-15], self-management tasks or appointment reminders [13,14,16], question and answer (Q&A) functions [17], and psychosocial support [17], some studies on digital health interventions in T1D have noted positive impacts on user acceptability [16], blood glucose monitoring behaviors [16], glycemic management [13,15], and clinic attendance [14,17]. Although promising, these results provide little insight into which of the multiple intervention components work to influence the desired outcomes, through which pathways, and under what circumstances [18]. The answer to this question is of relevance to health service researchers who are interested in intervention fidelity (eg, whether the intervention is implemented as intended) and policy makers who seek to understand how to maximize resource allocation amid growing financial constraints (eg, what elements are most effective and which groups are most likely to benefit) [19].

The UK Medical Research Council provides a framework for process evaluation that evaluates complex interventions by diving into the implementation process to understand intervention components, mechanisms of action, and the influencing context [20]. Realist evaluation, a specific type of process evaluation, is an explicit theory-driven evaluation framework that unveils the black box of the implementation process by specifying the context-intervention- mechanism-outcome (CIMO) configurations that could explain which components work (intervention and outcome), through which pathways (mechanisms), and for whom (contexts) [21,22]. In this study, we embedded a qualitative realist evaluation alongside a randomized controlled trial (RCT) that is investigating the effects of a tailored, multicomponent, text message–based digital health intervention (Keeping in Touch; KiT) on the transition experiences among emerging adults living with T1D. KiT integrates a broad range of validated intervention components, including educational content [4,12,13,15,23-26], transition coordination [17,27], appointment reminders [13,16,17], and psychosocial support [17], which have been delivered in both conventional structured programs and mobile-based interventions. By leveraging mobile technology, KiT overcomes the scalability challenges faced by conventional structured programs [28]. In addition, KiT stands out by offering more personalized content than many other mobile-based interventions [9]*,* tailoring information based on users’ needs and interests, and providing customized answers through its Q&A feature. The complete realist evaluation protocol (including the hypothesized CIMO configurations) [29] and KiT intervention development process [30] have been published previously.

### Objectives

The objectives of this qualitative realist evaluation were to (1) qualitatively examine the hypothesized CIMO configurations outlined in the initial program theory to validate whether and how they may be activated by KiT and (2) refine the initial program theory based on insights from the intervention implementation process.

## Methods

### Initial Program Theory

Combining the insights gained from the KiT program documentation and research articles on behavioral science theories for digital behavior change interventions, we previously identified 5 key KiT intervention components and 5 key intervention features, respectively [29]. These include the following:

1. Intervention components include T1D self-management information and suggestions, transition support information (ie, care navigation topic), problem-solving support (ie, Q&A), stress management strategies (ie, coping with T1D educational topic), and transition reminders (ie, appointment, test reminders, and note keeping)
2. Intervention features include credible sources, personalization, user-friendly message tone, real-time interactivity, and content format

By incorporating capability, opportunity, motivation, behavior model [31]; health action process approach [32]; and technology acceptance model [33] frameworks, we developed the initial program theory delineating the potential relationships among individual-level context, intervention components and features, mechanisms, and outcomes [29]. Therefore, we generated 10 hypotheses to understand which KiT intervention components and features may work, for which outcomes, and by which mechanism (Textbox 1).

**Initial program theory hypotheses and the refined hypotheses.**

**Research question: What may influence user engagement with Keeping in Touch (KiT)?**

- Initial hypothesis 1: 5 intervention components and 5 intervention features (intervention) may work together to shape user engagement (outcome) via perceived usefulness (mechanism).
- Refined hypothesis 1: 5 intervention components (intervention) and 5 intervention features (intervention) may foster perceived usefulness (mechanism), which may contribute to user engagement (outcome).
- Initial hypothesis 2: 5 intervention components and 5 intervention features (intervention) may work together to shape user engagement (outcome) via perceived ease of use (mechanism).
- Refined hypothesis 2: 4 intervention features (intervention), including personalization, content format, user-friendly message tone, and real-time interactivity, may foster perceived ease of use (mechanism), which may contribute to user engagement (outcome).

**Research question: Which KiT intervention components may influence participant outcomes and how?**

- Initial hypothesis 3: type 1 diabetes (T1D) self-management information and suggestions (intervention) may contribute to improved outcomes by supporting participants in gaining new knowledge and skills (mechanism).
- Initial hypothesis 4:
  T1D self-management information and suggestions (intervention) may contribute to improved outcomes by enhancing participants’ outcome expectancies (mechanism; belief about the consequence of performing a specific behavior).
- Refined hypotheses 3 and 4: T1D self-management information and suggestions (intervention) may contribute to improved self-reported blood glucose self-monitoring behaviors (outcome), transition readiness (outcome), or glycemic management (outcome), through the parallel mechanisms of knowledge (mechanism) and reinforcement (mechanism). These mechanisms may, in turn, shape outcome expectancies (mechanism), self-efficacy (outcome), negative emotions (ie, diabetes distress [outcome] and behavioral cueing [mechanism]). These pathways are more likely to be activated among those who are newly diagnosed (duration of diabetes [context]) or have limited baseline perceived access to information (context).
- Initial hypothesis 5: Transition support information (intervention) may support participants in gaining new knowledge and skills (mechanism).
- Refined hypothesis 5: Transition support information (intervention) may contribute to improved transition readiness (outcome) through 3 main mechanisms, namely knowledge (mechanism), outcome expectancies (mechanism), and reinforcement (mechanism). For individuals experiencing baseline diabetes distress (context) due to limited knowledge, KiT may help reduce negative emotions (outcome) and enhance self-efficacy (outcome) by addressing gaps in knowledge (mechanism) and outcome expectancies (mechanism).
- Initial hypothesis 6: Problem-solving support (intervention) may support participants in gaining new knowledge and skills (mechanism).
- Refined hypothesis 6: Facilitated by intervention features (intervention), including real-time interactivity, personalization, and credible sources, problem-solving support (intervention) may provide participants with knowledge (mechanism). Its use may be shaped by key contextual factors relating to the individual, such as baseline perceived knowledge or access to information (context) and awareness of available intervention components (context).
- Initial hypothesis 7 (not supported): Problem-solving support (intervention) may help participants enhance their self-efficacy (outcome).
- Initial hypothesis 8: Stress management strategies (intervention) may mitigate participants’ negative emotions (outcome).
- Refined hypothesis 8: Stress management strategies (intervention) may contribute to improved self-reported blood glucose self-monitoring behaviors (outcome) or transition readiness (outcome) by helping reduce negative emotions (outcome). These effects may be more relevant for individuals who are more recently diagnosed with diabetes (duration of diabetes [context]) or who have higher baseline diabetes distress (context) and lower baseline self-efficacy (context).
- Initial hypothesis 9 (not supported): Stress management strategies (intervention) may enhance participants’ self-efficacy (outcome).
- Initial hypothesis 10: Transition reminders (intervention) may work as a behavioral cueing mechanism for target behaviors.
- Refined hypothesis 10: Transition reminders (intervention) may facilitate improved transition readiness (outcome) by supporting behavioral cueing (mechanism) and enhancing self-efficacy (outcome), especially for those with strong competing priorities (context) and poorer memory ability (context).

### Study Setting

The multicenter KiT trial is ongoing and is being conducted at 6 pediatric diabetes clinics in Canada—4 in Ontario and 2 in Quebec [30]. The multicenter trial was launched in January 2023 and finished participant recruitment in September 2024, with a 12-month intervention duration and an anticipated trial end date in September 2025. Details about the KiT trial design are available in the published protocol [30].

### Ethical Considerations

Ethics approval for this embedded realist evaluation was obtained from the Trillium Health Partners Research Ethics Board (ID 1086). Ethics for the broader KiT trial (trial registration number NCT05434754) was obtained from Clinical Trials Ontario through the Hospital for Sick Children Research Ethics Board (project ID 3986) and McGill University Health Centre Research Ethics Board (project number MP-37-2023-8823).

Participants provided written informed consent for the embedded realist evaluation during the trial enrollment. The research team contacted the KiT trial participants who provided consent with a letter of information and an invitation to participate in an interview. Participants received a CAD $25 (US $18.25) electronic gift card to complete the interview. Identifiable data were stored in password-protected files on secure servers and were only accessible by approved research personnel. Data deidentification was performed before analyses. This paper follows the reporting requirements outlined by the Realist and Meta-narrative Evidence Synthesis: Evolving Standards (RAMESES) II checklist (Multimedia Appendix 1) [34].

### Participant Recruitment

Participants were eligible for this secondary study (ie, embedded realist evaluation) if they met the following criteria: (1) were randomized to the intervention arm with completed baseline surveys, (2) had a minimum of 3 months of intervention use to allow them to receive the 2 optional educational topics (based on users’ needs and interests, respectively [29]), and (3) confirmed receipt of ≥2 text messages from KiT in the month before the interview to mitigate potential recall bias. These criteria allowed us to include participants who were sufficiently familiar with the intervention. We used purposive sampling to facilitate representativeness across participants’ sociodemographic characteristics and intervention use. We recruited eligible participants until we achieved sufficient information power, which aims at offering new contextualized insights that contribute to current knowledge [35]. This approach allowed us to answer our research questions and refine the initial program theory to inform future testing in the full trial cohort.

### Data Collection Methods, Tools, and Procedures

#### Overview

We analyzed intervention use data to inform online semistructured realist interviews [36,37] exploring participants’ experiences engaging with KiT. We also extracted basic demographic data from the RCT baseline survey to understand the demographic characteristics of the interview participants.

#### Intervention Use

Intervention data were collected by Memotext, a third-party company that delivers and manages the KiT intervention. The Memotext system logs all incoming and outgoing messages from KiT, covering all intervention components, corresponding participant responses, and initiated messages. Because user engagement is multidimensional and extends beyond just technology use [38,39], we assessed it using several indicators, focusing on two aspects: (1) active engagement, measured by the response rate for standardized questions (ie, a set of 4 questions about baseline settings and optional topics that were asked uniformly to all participants at the beginning of the intervention, regardless of their specific needs), the total response rate at the time of interview (contained both standardized questions and optional questions that varied based on participants’ response to a readiness to transition questionnaire and questions about the topics they chose), and the use of available (optional) KiT components (including appointment reminder, test reminder, note keeping, and Q&A functions) and (2) passive engagement [38,40], assessed by the number of intervention topics received, which served as a proxy for sustained exposure to the educational information (delivered sequentially) and indirect cognitive engagement (where users may absorb information even without directly responding) [41].

#### Realist Interviews

We used semistructured realist interviews, guided by the “teacher-learner cycle” [37], a recommended method for data collection in realist evaluations [42]. Unlike conventional semistructured interviews, which focus on participants’ general perceptions [43], realist interviews generate insights about how the intervention is expected to work, in the form of hypothesized CIMO configurations [37].

The interviewer acted as a teacher by first introducing the hypothesized CIMO configurations to the participant, after which the participant acted as a teacher to confirm, deny, or modify the hypotheses based on their experiences [36]. For example, the interviewer would introduce CIMOs by stating the following [29]:

The first is educational information about diabetes management, transition from pediatric to adult care, and the Q&A. We think it helps participants gain more knowledge and skills, which helps them be better prepared for daily self-management and care transition. Does this align with your experiences? Can you provide some examples?

The realist interview guide (Multimedia Appendix 2) was developed based on initial CIMO configurations and related hypotheses described in detail in the study protocol [29]. It was piloted and iteratively refined during the data collection process with the input from 3 team members (LD, RW, and BP). Interviews were conducted by a single team member (RW) with no previous relationship with any participant or the T1D community and were audio recorded and transcribed verbatim by an independent third party.

#### Demographic Information

As part of the KiT trial, participants completed surveys at baseline, 6 months, and 12 months. We extracted participants’ baseline demographic data, including age, sex, ethnicity, and insurance information.

### Data Analysis

We performed a multistep qualitative data analysis following Gilmore et al [44], including interview data coding, case creation, and CIMO refinement.

#### Interview Data Coding

We developed the codebook according to the predefined constructs as per our objective to test and refine the initial program theory. Using a thematic analysis approach [45,46], we started deductive coding to capture the key constructs of the initial program theory with special attention paid to intervention components and features, mechanisms, and outcomes. We applied inductive coding when the additional emerging constructs did not fit within the predefined codes (such as context) and refined the codebook iteratively.

#### Case Creation

We integrated interview data with the participants’ demographic data and intervention use data (a proximal outcome) to facilitate a contextualized understanding of each participant’s case.

#### CIMO Refinement

We compared interview data with the hypothesized CIMO configurations in the initial program theory [19,47], which included 10 hypothesized pathways (Table 1). To achieve this, we first coded CIMO configurations in each transcript. CIMO elements were coded when elements occurred in a dyad (eg, intervention component → mechanism), triad (eg, intervention component → mechanism → outcome), or the full CIMO configuration [48]. We then created a list of CIMO configurations validated (or refined) at the participant level. As a final step, we synthesized the data across transcripts to identify patterns to support refinement of the program theory [49].

To ensure the credibility of the interview data analysis, 2 members of the research team (RW and BP) independently coded the first 8 transcripts and discussed all discrepancies until agreement was reached. Once a revised codebook was established, 1 team member (RW) coded all remaining transcripts, meeting regularly with other team members (LD and BP) to review and discuss the findings to collectively refine the program theory. No additional participant validation was conducted as this was incorporated as part of the realist interview approach. We used MAXQDA (VERBI Software) [50] to analyze qualitative data and R (R Foundation for Statistical Computing) and Microsoft Excel to triangulate data from different sources, create case constructions, and perform descriptive analyses to explore participant demographic characteristics.

### Positionality Statement

This interdisciplinary implementation study reflected a collaborative effort among team members with diverse expertise and perspectives, including implementation science researchers; clinical scientists in endocrinology, pediatrics, and pediatric psychology; as well as an emerging adult patient partner with lived experience of T1D. We took part in this study based on a shared commitment to improving the accessibility and relevance of interventions that address the multifaceted and evolving needs of emerging adults living with T1D.

Throughout data analysis and interpretation, the research team considered their own positionalities and professional identities. The reflexive discussions and cross-checked interpretations reduced individual biases and enhanced the credibility and depth of our findings. This approach allowed us to generate more comprehensive, context-sensitive, and rigorous insights.

## Results

### Participant Characteristics and Intervention Engagement

A total of 26 participants were invited to partake in the interview. Among them, 8 (31%) potential participants did not respond, and 2 (8%) agreed to participate but were lost to contact (ie, did not respond to follow-up messages to schedule the interview), leaving 16 (62%) participants recruited between July 2023 and May 2024. The interviews lasted 24 to 68 (mean 51, SD 10.4) minutes.

The 16 participants were aged 17 to 19 (median 17.9, IQR 17.8-18.1) years; 8 (50%) self-reported their sex as female and 8 (50%) as male. A total of 6 (38%) participants identified as White (with other major ethnic groups, including South Asian, Black, and Middle Eastern), and approximately two-thirds (n=10, 62%) of the participants had private insurance (Table 1; comparison with other intervention-arm participants who provided consent to participate in our study is available in Multimedia Appendix 3). Most participants responded to all 4 standardized questions and had a response rate of >80% to all questions sent by KiT from enrollment until their interview. Most participants received ≥4 educational topics, with driving, exercise, and nutrition and carbohydrates as the 3 most popular optional educational topics. Variability was observed in the use of optional KiT components, as expected, with a total of 12 (75%) participants using the appointment reminders, followed by the test reminders (n=7, 44%) as well as the note keeping and Q&A (n=2, 12%).

**Table 1:** Participant demographic characteristics and intervention engagement.

ID	Age (y)	Sex	Ethnic identity	Private insurance	Intervention duration at time of interview (wk)	Response rate for standardized questions^a^ (%)	Total response rate^b^ (%)	Number of topics received	Optional topic	Appointment reminder use	Test reminder use	Note-keeping use	Q&A^c^ use
ID01	17.9	Female	South Asian	No	31	100	92.3	8	Nutrition and carbohydrates	Yes	Yes	No	No
ID02	17.9	Female	East Asian	Yes	21	100	83.3	5	Drugs and alcohol	Yes	Yes	Yes	No
ID03	17.7	Female	South Asian	Yes	20	75	66.7	4	Driving	Yes	No	Yes	Yes
ID04	18.1	Female	White	Yes	19	75	81.8	5	Exercise	Yes	No	No	No
ID05	18.0	Male	White	Yes	15	100	100	4	School and work accommodations	No	No	No	No
ID06	17.6	Male	Black	Do not know	41	100	88.9	9	Nutrition and carbohydrates	No	No	No	No
ID07	17.9	Male	Middle Eastern	Yes	29	100	81.8	7	Nutrition and carbohydrates	Yes	Yes	No	Yes
ID08	17.8	Female	White	Yes	24	100	71.4	5	School and work accommodations	Yes	No	No	No
ID09	18.4	Male	Black	Do not know	36	100	66.7	5	Sexual health	Yes	Yes	No	No
ID10	17.7	Female	White	Do not know	21	100	100	6	Drugs and alcohol	Yes	Yes	No	No
ID11	17.8	Male	South Asian	Yes	40	100	88.9	6	Driving	No	No	No	No
ID12	18.8	Female	Southeast Asian	Yes	26	100	66.7	7	Exercise	Yes	No	No	No
ID13	18.0	Female	Middle Eastern	No	43	100	77.8	6	Exercise	Yes	Yes	No	No
ID14	17.8	Male	White	Yes	19	100	100	3	Driving	Yes	No	No	No
ID15	19.1	Male	Portuguese	Yes	23	100	77.8	1	Insulin adjustments	Yes	Yes	No	No
ID16	17.8	Male	White	Do not know	40	75	71.4	4	N/A^d^	No	No	No	No

^a^A set of 4 standardized questions that were asked uniformly to all participants at the beginning of the intervention, regardless of their specific needs.

^b^Contains both standardized questions and optional questions that varied based on participant responses to a readiness to transition questionnaire and questions about the topics they chose.

^c^Q&A: question and answer.

^d^N/A: not applicable.

### Revised Program Theory

#### Overview

**Figure 1:**
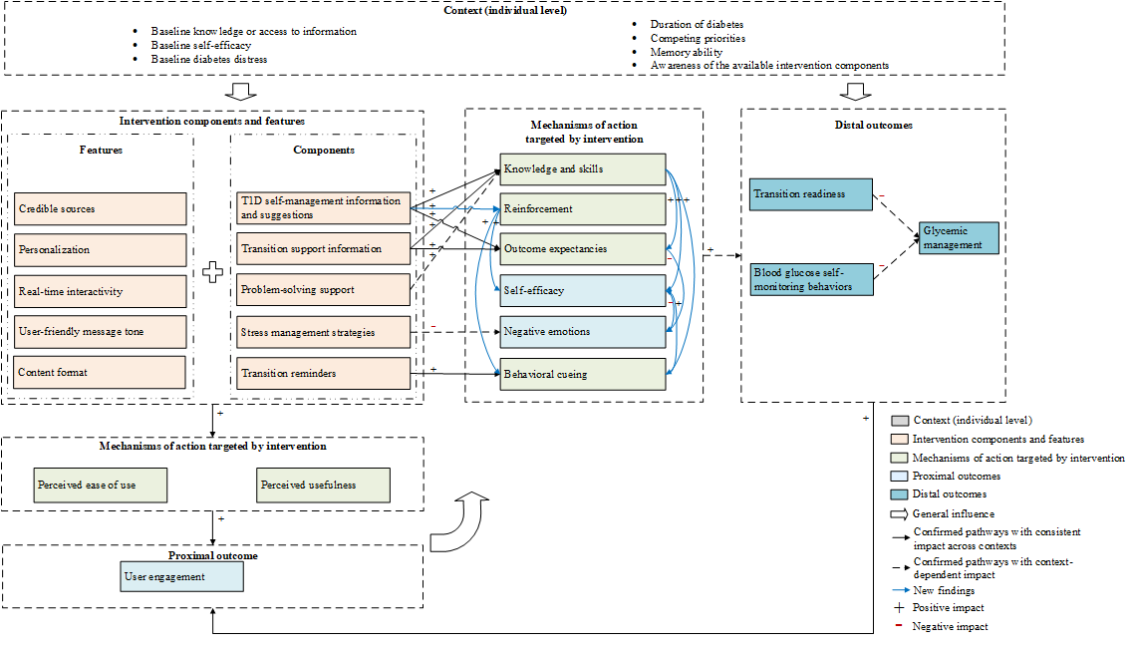
Refined program theory. T1D: type 1 diabetes.

The initial and refined hypotheses are reported in Textbox 1, with the revised program theory further elaborated in Figure 1. A breakdown of hypothesis test results by participant and their associated CIMO configurations can be found in Multimedia Appendices 4 and 5, respectively. Of the 10 initial hypotheses, 8 were congruent with participants’ experiences and further refined based on their feedback, whereas 2 were unsupported by this study’s findings. In addition, we found 1 new potential mechanism of influence with several associated pathways that could be incorporated into existing hypotheses. Across participants, consistent self-reported changes in the desired direction were noted for *knowledge*, *self-efficacy*, and *transition readiness*.

#### Understanding What Intervention Components May Drive Engagement

##### Overview

As anticipated, all 5 key intervention components were perceived as useful, with *T1D self-management information and suggestions* as the most frequently mentioned and highly rated component. Other intervention features, such as *credible sources*, *personalization* (ie, flexibility to choose topics), and *content format* (ie, attaching website links to messages to provide more information as needed), were cited as useful in conjunction with other intervention components:

Yes, and just the good amount of information, it’s not too much but not too little at the same time. So that’s another helpful part. And then also the links to provide more information if needed is also another good part of it. So yes.

ID06

Similarly, participants did not require extra effort to process the information delivered by the KiT intervention, and, consequently, they found it easy to use because of its intervention features, especially *personalization* (ie, flexibility to set frequency and time window of receiving educational messages), *message tone*, and *content format* (ie, attaching keywords to the beginning of the educational messages):

I feel like because they are—they are preferenced [sic] to our own—we can choose our own time windows that we can kind of get these messages. I like that because it doesn’t spam me with messages every single point of the day. So that’s a good thing.

ID13

Together, these findings supported refinements to the initial hypotheses 1 and 2, as outlined subsequently and in Figure 1.

##### Refinement to Hypothesis 1

Five intervention components (intervention) and 5 intervention features (intervention) may foster perceived *usefulness* (mechanism), which may contribute to *user engagement* (outcome).

##### Refinement to Hypothesis 2

Four intervention features (intervention), including *personalization*, *content format*, *user-friendly message tone*, and *real-time interactivity*, may foster perceived *ease of use* (mechanism), which may contribute to *user engagement* (outcome).

#### Understanding What Mechanisms May Drive Improvement in Outcomes

##### Providing T1D Self-Management Information Was Perceived to Activate Multiple Mechanisms and Drive Change in Multiple Outcomes

###### Overview

All participants described that *T1D self-management information and suggestions* (intervention) meaningfully increased their *knowledge* (mechanism), providing new facts or deeper awareness. Some participants further demonstrated that this facilitated their self-reported *blood glucose self-monitoring behaviors* (outcome), contributed to improved *glycemic management* (outcome), or helped them better prepare for transition to take full responsibility for their T1D management (*transition readiness* [outcome]). Across participants, *T1D self-management information and suggestions* (intervention) was perceived to influence self-management through a chain of mechanisms, starting from *knowledge* (mechanism) to influence *outcome expectancies* (mechanism), *self-efficacy* (outcome), *negative emotions* (ie, diabetes distress [outcome]), and *behavior cueing* (mechanism). For example, 7 (44%) of the 16 participants noted that the T1D information improved their understanding of the positive and negative consequences of T1D self-management (*outcome expectancies* [mechanism]), increasing motivation to perform daily T1D self-management. Some participants also demonstrated a positive feedback loop, where perceived positive change in health outcomes (eg, *glycemic management*) further reinforced their motivation and intervention engagement:

Before I didn’t know how to do something and I didn’t want to do it. So now because I do know how to maintain it, and I know the consequences and I know how to or ways to kind of improve it, improve my diabetes management, my glucose readings, I kind of want to do it better...Yes, because I know the positives and the negatives. The negatives and the positives kind of work together in motivating because knowing the negatives is something that you don’t want to happen to you, but the positives is something that you do want to ensure.

ID13

And also, the outcome expectancies, I think it helps you because it helps you with your self-management and self-confidence. And I feel like that, overall, just helps your blood sugar actually go down in real life. So then because your blood sugar actually went down, you kind of feel like it’s actually helping and your outcome in the end will actually be better, right, because it’s helping with your self-monitoring, and you’re actually seeing results.

ID05

In addition, most participants identified a parallel mechanism in which *T1D self-management information and suggestions* (intervention) reinforced existing knowledge (*reinforcement* [mechanism]), reminding them of information they had previously learned but set aside, thereby supporting *blood glucose self-monitoring behaviors* (outcome). Some suggested that the effectiveness of this intervention component may depend on individual characteristics, such as *duration of diabetes* (context) and *baseline perceived access to information* (context). Specifically, those who are newly diagnosed (*duration of diabetes* [context]) or have perceived limited access to credible information (*baseline perceived access to information* [context]) may gain more new *knowledge* (mechanism) and therefore benefit more from the KiT *T1D self-management information and suggestions* (intervention):

I guess the knowledge and skills aspect. The KiT gives a lot of good and really helpful information. Especially about hypo and hyper glycemia. Especially when you’re first and newly diagnosed I think those are the two most important things to pay attention to first. As you get older, things like travelling and driving would also be something to look into. I think just having that knowledge before it happens would be very helpful.

ID12

Together, these findings supported combining the initial hypotheses 3 and 4 and refining the mechanisms, as outlined subsequently and in Figure 1.

###### Refinement to Hypotheses 3 and 4

T1D self-management information and suggestions (intervention) may contribute to improved self-reported blood glucose self-monitoring behaviors (outcome), transition readiness (outcome), or glycemic management (outcome) through the parallel mechanisms of knowledge (mechanism) and reinforcement (mechanism). These mechanisms may, in turn, shape outcome expectancies (mechanism), self-efficacy (outcome), negative emotions (ie, diabetes distress [outcome]), and behavioral cueing (mechanism). These pathways are more likely to be activated among those who are newly diagnosed (duration of diabetes [context]) or have limited baseline perceived access to information (context).

#### Transition Support Information Was Perceived to Contribute to Transition Readiness Through Multiple Mechanisms

##### Overview

A total of 7 (44%) of the 16 participants described the positive influence of *transition support information* (intervention) either by providing new *knowledge* (mechanism) or reinforcing existing knowledge (*reinforcement* [mechanism]), both of which improved their ability to navigate the health care system (*transition readiness* [outcome]). Some participants highlighted a chain of mechanisms whereby *outcome expectancies* (mechanism) linked *knowledge* (mechanism) to *transition readiness* (outcome). For participants experiencing *baseline* *diabetes distress* (context) due to limited knowledge about care transitions or T1D management more general, *transition support information* (intervention) helped reduce their diabetes distress (*negative emotions* [outcome]) by increasing *knowledge* (mechanism) and fostering a sense of preparedness (*self-efficacy* [outcome]):

I think it was pretty recently, actually. And just giving tips about how to reach certain care teams when I need the support. You know, this is what you can expect, certain things like that. And I did find them pretty helpful. As much as I do feel very prepared from my pediatric team, they also don’t know everything that does go on in the [world of adult care] because they obviously don’t work there. So, I did find it helpful when they did give specific transition tips.

ID04

Together, these findings supported refining hypothesis 5, as outlined subsequently and in Figure 1.

##### Refinement to Hypothesis 5

Transition support information (intervention) may contribute to improved transition readiness (outcome) through 3 main mechanisms, namely knowledge (outcome), outcome expectancies (mechanism), and reinforcement (mechanism). For individuals experiencing baseline diabetes distress (context) due to limited knowledge, KiT may help reduce negative emotions (outcome) and enhance self-efficacy (outcome) by addressing gaps in knowledge (mechanism) and outcome expectancies (mechanism).

#### The Perceived Impact of Problem-Solving Support and Stress Management Strategies May Depend Heavily on Context

##### Problem Solving Support

Problem-solving support (intervention) was perceived to help increase knowledge (mechanism), supported by key intervention features, including real-time interactivity (intervention), personalization (intervention), and credible sources (intervention). Most participants reported not using the problem-solving support (intervention) as they did not have a specific question in mind (baseline perceived knowledge or access to information [context]) or were unaware of this intervention component (awareness of available intervention components [context]):

Oh. So no I haven’t done that yet. So I might actually do that now after the interview, but yes, no I haven’t done that yet...I’m going to be honest, I don’t—I didn’t know it existed.

ID05

These insights were congruent with the initial hypothesis 6 but not congruent with hypothesis 7 in this study’s sample due to low engagement with this component. We further refined hypothesis 6 as mentioned subsequently.

##### Refinement to Hypothesis 6

Facilitated by intervention features (intervention), including *real-time interactivity*, *personalization*, and *credible sources*, *problem-solving support* (intervention) may provide participants with *knowledge* (mechanism). Its use may be shaped by key contextual factors relating to the individual, such as *baseline perceived knowledge or access to information* (context) and *awareness of available intervention components* (context).

##### Stress Management Strategies

Participants described how *stress management strategies* (intervention) helped reduce their diabetes distress (*negative emotions*, [outcome]) by reminding them to change their negative mindset, confirming hypothesis 8. A total of 3 (19%) of the 16 participants further linked the pathway to increased *blood glucose self-monitoring behaviors* (outcome) and/or *transition readiness* (outcome):

One great example, especially during exam time, was just stress from school because obviously it’s a very stressful time having to study for all these final exams, having to write them. It’s very stressful. The last few weeks have been very hard to be able to sit down, study 12/13 hours a day, then go to sleep, wake up, do the same thing. So, I feel like the tips that they gave were pretty useful when it came to helping with that...One that I can remember was breathe in, breathe out I believe, just sitting there, just taking a moment of mindfulness just to breathe in, breathe out, help release some of that...So, obviously, when you’re stressed, you’re not going to pay attention as much to anything but studying. So, when I was breathing in, breathing out, that also helped me remind to check on my blood sugar to make sure that it’s in a proper place and to make sure that it’s in a good range and stuff.

ID11

In the meantime, the effectiveness of this pathway may depend on 3 individual characteristics, including *duration of diabetes* (context), *baseline diabetes distress* (context), and *baseline self-efficacy* (context). For instance, 2 (12%) of the 16 participants found *stress management information* (intervention) less impactful because they did not feel a strong need to address diabetes distress (*negative emotions* [outcome]) because of either having already developed effective coping strategies and *self-efficacy* (context) or becoming used to living with T1D due to the long *duration of diabetes* (context):

I’ve had type 1 diabetes from a very young age, so I’ve always lived with it, and so I never really had problems coping with it, so I would say that those never really applied to me too much because I’ve never really felt that I need help to cope with it since it’s always just been with me my whole life.

ID07

In contrast to the direct influence on *negative emotions* (outcome), our findings were not congruent with hypothesis 9, which hypothesized that *stress management information* (intervention) would positively influence *self-efficacy* (outcome). Hypothesis 8 was refined based on insights around individual context.

##### Refinement to Hypothesis 8

Stress management strategies (intervention) may contribute to improved self-reported blood glucose self-monitoring behaviors (outcome) or transition readiness (outcome) by helping reduce negative emotions (outcome). These effects may be more relevant for individuals who are more recently diagnosed with diabetes (duration of diabetes [context]) or who have higher baseline diabetes distress (context) and lower baseline self-efficacy (context).

#### Transition Reminders Were Perceived to Have a Clear and Straightforward Impact

##### Overview

We found congruence in our data for hypothesis 10, with most participants reporting that the *transition reminders* (intervention) cued appointment attendance by preventing scheduling conflicts (*behavioral cueing* [mechanism]), ultimately facilitating the transition to independent T1D self-management (*transition readiness* [outcome]). This mechanism was especially significant for those with coexisting (and strong) *competing priorities* (context) and challenges with *memory ability* (context). Some participants further described the potential chain of mechanistic pathways where *transition reminders* (intervention) enhanced *self-efficacy* (outcome), which in turn improved *transition readiness* (outcome) as a result:

I’m not very good at remembering things so having the constant reminders of getting tests done or preparing for the appointments and the actual appointment itself was very useful...So, I had an appointment yesterday that I put into the system and I like forgot about it a couple of times, like in the month leading up to it. And I was like, “Oh, right. I have that. So, OK, I’ve got to take the day off work. I’ve got to make sure that I’ve got everything set up for it.” And then I’ll forget about it again, then it will come back up on my phone and I’ll be like, “Oh, right. That’s that day. OK, I can’t do anything that day.”

ID14

Reminding myself well in advance that I have an appointment increases my confidence because I have—me remembering I can do this. Now, I can remember I need to do this and I have these questions and I can go in feeling more prepared for an appointment.

ID04

From these insights, the initial hypothesis 10 was refined as demonstrated in Figure 1.

##### Refinement of Hypothesis 10

*Transition reminders* (intervention) may facilitate improved *transition* *readiness* (outcome) by supporting *behavioral cueing* (mechanism) and enhancing *self-efficacy* (outcome), especially for those with strong *competing priorities* (context) and poorer *memory ability* (context).

## Discussion

### Principal Findings

Qualitatively developing and synthesizing participant-specific CIMO configurations provided in-depth context into what KiT intervention features were being used, by whom, and via what mechanism. Taken together, these insights provided intervention-level insights into the different ways KiT may work, for whom, and how. Specifically, we found the following:

1. All 5 intervention components may add value but to varying degrees. T1D self-management information and suggestions were perceived to have a universally positive impact across all participants, whereas the perceived usefulness of other components appeared to vary depending on the participant.
2. The KiT intervention may operate via multiple chains of interconnected mechanisms (rather than parallel chains of independent mechanisms), which appear to start with providing new knowledge or reinforcement of existing knowledge.
3. All participants reported experiencing some benefit from KiT, although the nature of perceived benefit and how it manifested varied across participants.

### Insights Into What May Work and for Whom

The importance of *educational content* (intervention) and *reminders* (intervention) as core intervention components aligns with previous research [51-53]. In contrast, the use of *problem-solving support* (intervention) may vary depending on participants’ *awareness of available intervention* *components* (context) and *perceived knowledge or access to information* (context). Similar challenges have been reported in previous studies [40,54], emphasizing the need for robust onboarding and routine reminders of available intervention components to improve engagement [40]. Similarly, the *stress management strategies* (intervention) are more likely to be effective for individuals with high *baseline diabetes distress* (context) and low confidence (*self-efficacy* [context]), reinforcing the value of personalized interventions that account for individual differences, such as duration of diabetes [55], baseline knowledge [56,57], and perceived needs [58].

These findings highlight the limitations of one-size**-**fits**-**all approaches, advocating for context-sensitive, multicomponent interventions that accommodate participant heterogeneity [57,59]. This requires identifying key subgroups, often described as archetypes [60], profiles [61], or personas [62]. Our results suggest that digital health interventions similar to KiT should consider tailored strategies for individuals with more recent diabetes diagnoses (*duration of diabetes* [context]), perceived limited access to credible knowledge (*baseline knowledge or access to information*, [context]), lower *baseline self-efficacy* (context), or higher *baseline diabetes distress* (owing to the recency of their diagnosis [context]). Tailored strategies could include educational content on T1D burnout and building coping strategies [63,64] and mood-enhancing messages (eg, self-affirmation, gratitude, and “mood booster”) [64]. In the meantime, further research is needed to better understand the specific needs of other subgroups of emerging adults living with T1D to inform targeted intervention design.

### Understanding How KiT May Work

Previous studies have demonstrated that *educational content* (intervention) can serve as valuable reminders to nudge self-management behaviors [51,56,65], aligning with our results that *reinforcement* (mechanism) of existing knowledge might be as important as sharing new *knowledge* (mechanism) [59]. Desveaux et al [66] and Sandborg et al [67] illustrated that digital health interventions increased participants’ understanding of their disease condition (ie, *knowledge* [mechanism]), which translated to improved motivation and *self-management* (outcome). Even when some of the information is already known, reinforcing it (*reinforcement* [mechanism]) may still enhance *self-management* (outcome) [56]. Our work builds on and integrates these findings by specifying how *knowledge* (mechanism), *reinforcement* (mechanism), *outcome expectancies* (mechanism)*,* *self-efficacy* (outcome), *negative emotion* (ie, diabetes distress [outcome]), and *behavioral cueing* (mechanism) may operate as chains of interconnected mechanisms. This interconnectedness of mechanisms reinforces the complexity of digital health interventions and advances our understanding of how their impact may be realized. By identifying *knowledge* (mechanism) and *reinforcement* (mechanism) as candidate upstream mechanisms, our results suggest that prioritizing these mechanisms is a high-yield consideration in designing digital health interventions to support chronic disease *self-management* (outcome). Taking it one step further, measuring *knowledge* (mechanism) as an upstream indicator of early impact may provide insight into whether a digital health intervention is well-positioned to achieve long-term goals (eg, *glycemic management* [outcome]).

### How Long KiT May Take to Work: Insights for the Broader RCT

Participants reported noticeable changes in mechanisms (*knowledge*) and proximal outcomes (*self-efficacy* and *negative emotions due to diabetes distres*s) but reported limited perceived impact on the distal outcome—*glycemic management* (outcome)*.* After ruling out technical problems with KiT’s intervention components design and delivery, we speculate that the lack of meaningful change in glycated hemoglobin (HbA_1c_) may be an artifact of timing or reflect a mismatch between interventions and desired outcomes. First, text message–based interventions may require more time to influence distal outcomes such as HbA_1c_ [53], and participants in this study were interviewed during the first half of their intervention period. Previous studies have similarly noted meaningful changes in *self-efficacy*, *negative emotions* (ie, diabetes distress), and *knowledge* within 3 to 6 months of intervention exposure [53,68-70] and up to as early as 8 weeks [68], suggesting that this time frame is a reasonable window for proximal outcomes. However, while meta-analyses have shown digital health interventions can positively impact HbA_1c_ [71,72], the literature remains variable. For instance, Dobson et al [73] observed a significant reduction in HbA_1c_ at 9 months, while Franklin et al [13] and Castensøe-Seidenfaden et al [74] failed to observe an effect at 12 months. Our results suggest that the broader RCT (12 mo) may provide a clearer understanding of the minimum intervention length needed to see changes in HbA_1c_. These findings reinforce the value of exploring upstream changes in the short term through realist evaluations.

Furthermore, while HbA_1c_ is widely adopted as a primary distal outcome in diabetes management interventions, previous literature has revealed its limitations in capturing short-term (<2 mo) changes in *glycemic management* and other important aspects such as quality of life [75]. These findings suggested the need to move beyond the reliance on HbA_1c_ alone. Furthermore, aligning with the broader KiT trial that incorporated additional outcomes, including *transition readiness* and *self-efficacy*, future research on similar interventions might benefit from incorporating additional outcomes, such as mean glucose levels [69], time-in range [75], and patient-reported quality of life [73], to provide a more comprehensive understanding of potential impact.

### Methodological Contributions to Implementation Science

This embedded, theory-driven design contributes to both explanatory theory-building and pragmatic intervention strategies, illustrating how realist evaluation can facilitate the interpretability and utility of RCTs in implementation science.

First, while RCTs offer an unbiased estimate of intervention effectiveness, they typically provide only overall treatment effects without unpacking the implementation black box of how specific components of a multicomponent intervention work [14,17,76]. Embedded alongside the RCT, our realist evaluation filled this knowledge gap by uncovering the mechanisms through which particular intervention components function. This deepens understanding of the intervention’s inner workings and supports a more theoretically grounded interpretation of the trial results [77,78], particularly valuable for complex digital health interventions such as KiT [20].

Second, recognizing that many RCTs yield suboptimal results in distal outcomes [79,80], we timed our data collection alongside the trial. This approach enabled exploration of upstream mechanisms and proximal outcomes, revealing a cascading effect of KiT in which early changes were initiated, although distal clinical effects were not yet fully observable. By capturing early-stage signals, this approach offers a more comprehensive and nuanced understanding of how the intervention works over time. In the meantime, it also prompts reflection on whether we are measuring the right outcomes at the right time, which is an important consideration for fair and meaningful evaluation [81].

Third, our realist evaluation identified context-intervention configurations that specify which groups of recipients may be most likely to benefit from which intervention components. This enhances the utility of RCT findings by enriching the answer to “Does it work?” with insights into “Which components work and for whom?” [20,78]. Consequently, it offers greater pragmatic value for real-world decision-making by informing targeted intervention refinement and supporting strategy development, particularly important in health systems where resources are limited and tailoring is essential for effective scale-up.

### Limitations

This realist evaluation study, embedded within a broader KiT trial, was voluntary, and 10 (38%) of the 26 participants we contacted did not respond. Most of the interview participants who responded had high engagement with KiT prompts. This introduces potential selection bias and limits the transferability of the findings to individuals with moderate or low engagement levels. While highly engaged participants may experience positive outcomes [82], those with lower engagement may not benefit as much from the intervention in terms of improving their knowledge and motivation for T1D self-management. This may be due to the lack of a positive feedback loop that reinforces engagement and behavior change, potentially leading to an absence of the desired outcome. To better support adolescents and young adults, who may benefit from this intervention the most, future research may consider the following: (1) enhancing implementation strategies that target modifiable predictors of user engagement, such as incorporating the persuasive design and behavior change techniques to strengthen user motivation [83], timing interventions in summer months to reduce competing priorities [84], and fostering a supportive environment (particularly through health care providers [85]) and (2) exploring the specific needs of individuals with moderate or low engagement, recognizing that engagement is shaped by an interplay between intervention functions and user needs [86]. Second, given the exploratory and qualitative nature of this work, our primary goal was to generate early insights into the hypothesized mechanisms of change through which KiT might operate (ie, to validate or refine CIMO configurations). Quantitative evaluations of effectiveness were beyond the scope of this study and the available data. Combining these descriptive findings with the forthcoming KiT trial results will provide a more comprehensive understanding of the intervention’s impact. Third, although we recognize the value of understanding the representativeness of the interview subsample compared to the broader sample of all RCT intervention-arm participants, conducting a full comparison of engagement levels and outcome data was beyond the scope of this study. Finally, while we focused on individual-level contextual factors, we acknowledge the need to explore broader system-level factors (eg, health care access and social norms) and how they interact to influence emerging adults’ transition to adult care experiences.

### Conclusions

The preliminary findings indicate that the KiT intervention is a promising intervention for supporting emerging adults living with T1D during their transition to adult care by enhancing their self-reported knowledge and motivation for self-management. T1D self-management information and suggestions were perceived as a universally valuable intervention component, suggesting it should remain a core component in interventions targeting this population. The KiT intervention also appears to operate through multiple pathways depending on specific users’ characteristics, supporting that digital health interventions should move beyond one-size-fits-all approaches to expect and allow users to tailor the intervention based on their needs, which is a competitive advantage that is enabled by their KiT’s digital construction. Future work that aims to support emerging adults living with T1D through behavioral interventions should focus on the unique needs of subgroups from the outset, including those who are recently diagnosed, have lower self-efficacy, or are experiencing diabetes distress, to tailor intervention components in line with individualized needs. This creates a user-friendly engagement environment more likely to enable sustained use over time. While improvements were observed in mechanisms and proximal outcomes (eg, knowledge and self-efficacy), changes in distal outcomes (eg, glycemic management) were less evident, necessitating the need for triangulation of these results from this study with the upcoming KiT trial findings.

## Appendix

Multimedia Appendix 1 Realist and Meta-Narrative Evidence Synthesis: Evolving Standards (RAMESES) II checklist.

Multimedia Appendix 2 Keeping in Touch (KiT) realist evaluation interview guides.

Multimedia Appendix 3 Comparison of demographic characteristics between interview participants and other intervention-arm participants who provided consent for this study.

Multimedia Appendix 4 Result matrix for each participant.

Multimedia Appendix 5 Detailed pathways and contexts of each participant.

## Abbreviations

CIMO: context-intervention-mechanism-outcome
HbA1c: glycated hemoglobin
KiT: Keeping in Touch
Q&A: question and answer
RAMESES: Realist and Meta-Narrative Evidence Synthesis: Evolving Standards
RCT: randomized controlled trial
T1D: type 1 diabetes

## Data Availability

The datasets generated or analyzed during this study are not publicly available as we do not have permission from the interview participants or the research ethics board to publish the raw data, which may lead to potential identification of participants, but the deidentified quotes are available from the corresponding author on reasonable request.
