# Supplementary material for: Insights Into How Digital Health Interventions Shape Outcomes for Emerging Adults Living With Type 1 Diabetes: Qualitative Realist Process Evaluation
Source: J Med Internet Res. 2025 Sep 5;27:e70401. doi: 10.2196/70401 (PMC12449673; doi:10.2196/70401)
Supplement: Multimedia Appendix 2 [file jmir_v27i1e70401_app2.docx]

### Appendix Table 2 KiT realist evaluation interview guides

### Interview Guide 1: Participants with high levels of engagement

**Part 1: General background**

1. **Context:** To start, can you tell me about your experiences managing your diabetes before KiT? What concerns you had, what you were confident about, and how you approached managing things?
2. **Context:** What resources do you use to help you manage your diabetes?
3. **Change in motivation:** You’ve mentioned (A, B, C) in your responses above. Has any of this shifted since you started interacting with KiT?

**Part 2: KiT engagement**

1. **Context:** What motivated you to try KiT in the first place (e.g., individual attitudes, previous experience with other T1D self-management tools)?
2. **KiT intervention:** Can you walk me through experiences with KiT so far?
3. **Outcome and mechanisms:** I can see that you have been interacting with KiT actively (according to intervention usage data). What aspects of KiT do you think worked well?
4. **Outcome and mechanisms:** We had a few ideas about how KiT would engage people and we think that it probably works differently for different people. One idea was that the content KiT sends is reliable and helpful. Another idea is that the information format and layout is clear and easy to use. What do you think about those ideas (e.g., educational information, appointment reminders, KiT message tone)?
5. **Context:** How does KiT compare to [other resources the participant mentioned]?

**Part 3: Distal outcomes**

1. **Outcomes:** What things do you think KiT will change for you (e.g., improved transition knowledge/skills, diabetes management outcomes)?

**At follow up**:

- 1. Can you give me an example of [outcome named in previous question]? Have you noticed any changes in [outcome named in previous question]?
  2. Do you think the outcomes have been the same for all participants? If not, in what ways have they been different (e.g., for those who are confident vs not confident about their own ability to manage diabetes)?

1. **Mechanisms:** We are also curious about how KiT helped to cause these changes in outcomes. What do you think happened that led to those changes?

**At follow up:**

This is how we think KiT works. We think that KiT helps to improve transition experiences mainly from three pathways.

1. The first is educational information about diabetes management, transition from pediatric to adult care and the Q&A. We think it helps participants gain more knowledge and skills, which helps them be better prepared for daily self-management and care transition. Does this align with your experiences? Can you provide some examples?
2. Second, KiT provides participants with motivation. For instance, information about diabetes management helps increase their belief about the consequence of using KiT, the Q&A helps improve their self-confidence in managing diabetes, and information about stress management also helps improve their self-confidence while addressing their negative emotions. Does this align with your experiences? Can you provide some examples?
3. Last, KiT acts as a reminder cueing routine diabetes management and appointment attendance. These three pathways help improve participants’ readiness to transit from paediatric to adult care, their self-blood glucose monitoring behaviour, and eventually, their blood glucose. Does this align with your experiences? Can you provide some examples?
4. **Other CIMOs:** What else do you think we need to know, to really understand how KiT has worked for you?

**Part 4: Intervention improvement**

1. What aspect(s) of KiT could we change to make it more effective?

**Prompts:**

1. *Content:* Any other information/resources you need to better manage diabetes?
2. *Delivery:* What can be done to better integrate this tool into your daily routine?
3. Is there anything else we could have done to better support you using KiT?
4. Is there anything else you would like to mention we haven’t discussed today?

### Interview Guide 2: Participants with low to medium engagement

**Part 1: General background**

1. **Context:** To start, can you tell me about your experiences managing your diabetes before KiT? What concerns you had, what you were confident about, and how you approached managing things?
2. **Context:** What resources do you use to help you manage your diabetes?
3. **Change in motivation:** You’ve mentioned (A, B, C) in your responses above. Has any of this shifted since you started interacting with KiT?

**Part 2: KiT engagement**

1. **Context:** What motivated you to try KiT in the first place (e.g., individual attitudes, previous experience with other T1D self-management tools)?
2. **KiT intervention:** Can you walk me through experiences with the KiT so far?
3. **Outcome and mechanisms:** We had a few ideas about how KiT would engage people and we think that it probably works differently for different people. One idea was that the content KiT sends is reliable and helpful. Another idea is that the information format and layout is clear and easy to use. We are curious why it did not work out (e.g., what were you hoping that KiT would offer that it didn’t)? Can you give me an example?
4. **Context:** How does KiT compare to [other resources the participant mentioned]?
5. **Other CIMOs:** What else do you think we need to know, to really understand how KiT has worked for you?

**Part 3: Intervention improvement**

1. What aspect(s) of KiT could we change to make it more effective?

**Prompts:**

1. *Content:* Any other information/resources you need to better manage diabetes?
2. *Delivery:* What can be done to better integrate this tool into your daily routine?
3. Is there anything else we could have done to better support you using KiT?
4. Is there anything else you would like to mention we haven’t discussed today?
