# Supplementary material for: Insights Into How Digital Health Interventions Shape Outcomes for Emerging Adults Living With Type 1 Diabetes: Qualitative Realist Process Evaluation
Source: J Med Internet Res. 2025 Sep 5;27:e70401. doi: 10.2196/70401 (PMC12449673; doi:10.2196/70401)
Supplement: Multimedia Appendix 3 [file jmir_v27i1e70401_app3.docx]

Appendix Table 3 Comparison on demographic characteristics between interview participants and other intervention-arm participants who consented to this study

| **Demographic characteristics** | **Interview participants (N=16)** | **Other participants**^a^ **(N=57)** | **Total (N=73)** |
| --- | --- | --- | --- |
|  |  |  |  |
| **Age,** Median (Q1, Q3) | 17.9 (17.8 ,18.1) | 18.1 (17.8 ,18.2) | 18.0 (17.8 ,18.2) |
| **Sex**^b^, n (%) |  |  |  |
| Female | 8 (50%) | 31 (55%) | 39 (54%) |
| Male | 8 (50%) | 25 (45%) | 33 (46%) |
| **Ethnic identity**^c^, n (%) |  |  |  |
| White | 6 (38%) | 32 (58%) | 38 (54%) |
| Middle Eastern | 2 (12%) | 9 (16%) | 11 (15%) |
| Asian (South, Southeast, East) | 5 (31%) | 1 (2%) | 6 (8%) |
| Black | 2 (12%) | 3 (5%) | 5 (7%) |
| Other | 1 (6%) | 10 (18%) | 11 (15%) |
| **Private insurance**, n (%) |  |  |  |
| Yes | 10 (62%) | 27 (47%) | 37 (51%) |
| No | 2 (12%) | 20 (35%) | 22 (30%) |
| Don't know | 4 (25%) | 10 (18%) | 14 (19%) |

N.B. ^a^: Other intervention arm participants who consented to this study. ^b^: Missing value for other intervention arm participants who consented to this study was 1. ^c^: Missing value for other intervention arm participants who consented to this study was 2.
