# Supplementary material for: Insights Into How Digital Health Interventions Shape Outcomes for Emerging Adults Living With Type 1 Diabetes: Qualitative Realist Process Evaluation
Source: J Med Internet Res. 2025 Sep 5;27:e70401. doi: 10.2196/70401 (PMC12449673; doi:10.2196/70401)
Supplement: Multimedia Appendix 4 [file jmir_v27i1e70401_app4.docx]

Appendix Table 4 Result matrix for each participant

| ID | H1 | H2 | H3 | H4 | H5 | H6 | H7 | H8 | H9 | H10 | Number of newly identified pathways |
| --- | --- | --- | --- | --- | --- | --- | --- | --- | --- | --- | --- |
|  |  |  |  |  |  |  |  |  |  |  |  |
| ID01 | Y | Y | Y | Y | N | N | N | Y | N | Y | 1 |
| ID02 | Y | Y | Y | Y | Y | N | N | N | N | Y | 1 |
| ID03 | Y | Y | Y | N | Y | N | N | Y | N | Y | 1 |
| ID04 | Y | Y | Y | Y | Y | N | N | N | N | Y | 1 |
| ID05 | Y | Y | Y | Y | Y | N | N | N | N | N | 1 |
| ID06 | Y | Y | Y | Y | Y | N | N | N | N | N | 1 |
| ID07 | Y | Y | Y | N | N | Y | N | N | N | N | 0 |
| ID08 | Y | Y | Y | N | Y | N | N | Y | N | Y | 1 |
| ID09 | Y | Y | Y | N | N | N | N | N | N | Y | 3 |
| ID10 | Y | Y | Y | Y | N | N | N | N | N | Y | 2 |
| ID11 | Y | Y | Y | N | N | N | N | Y | N | N | 1 |
| ID12 | Y | Y | Y | N | N | N | N | Y | N | Y | 2 |
| ID13 | Y | Y | Y | Y | N | N | N | N | N | N | 1 |
| ID14 | Y | Y | Y | N | N | N | N | N | N | Y | 0 |
| ID15 | Y | Y | Y | N | N | N | N | Y | N | Y | 1 |
| ID16 | Y | Y | Y | N | N | N | N | N | N | N | 0 |

N.B. H: Hypothesis, details could be found in Table 1. Details of newly identified pathways could be found in Appendix Table 5.
