# Supplementary material for: Insights Into How Digital Health Interventions Shape Outcomes for Emerging Adults Living With Type 1 Diabetes: Qualitative Realist Process Evaluation
Source: J Med Internet Res. 2025 Sep 5;27:e70401. doi: 10.2196/70401 (PMC12449673; doi:10.2196/70401)
Supplement: Multimedia Appendix 5 [file jmir_v27i1e70401_app5.docx]

Appendix Table 5 Detailed pathways and contexts of each participant

| ID | Baseline personal contexts | Interview hypotheses and validated code configurations |
| --- | --- | --- |
| ID01 | - Long duration of diabetes - Perceived strong need for knowledge - With diabetes stress | - **H1:** T1D self-management information and suggestions (I) +/ Transition reminders (I) +/ Stress management strategies (I) +/ Personalization (I) +/ Content format (I) +/ User-friendly message tone (I) 🡪 Perceived usefulness (M) - **H2:** T1D self-management information and suggestions (I) +/ Personalization (I) 🡪 Perceived ease of use (M) - **H3:** T1D self-management information and suggestions (I) 🡪 Knowledge and skills (M) 🡪 Self-efficacy (M) 🡪 Negative emotions (M) 🡪 Blood glucose self-monitoring behaviours (O) - **H4:** T1D self-management information and suggestions (I) 🡪 Outcome expectancies (M) - **H8:** Stress management strategies (I) 🡪 Reinforcement (M) 🡪 Negative emotions (M) - **H10:** Competing priorities (C) + Transition reminders (I) 🡪 Behavioural cueing (M) + Self-efficacy (M) 🡪 Transition readiness (O) - **New:** T1D self-management information and suggestions (I) 🡪 Reinforcement (M) 🡪 Blood glucose self-monitoring behaviours (O) |
| ID02 | - Short duration of diabetes | - **H1:** T1D self-management information and suggestions (I) +/ Transition reminders (I) +/ Transition support information (I) +/ Personalization (I) +/ User-friendly message tone (I) 🡪 Perceived usefulness (M) - **H2:** T1D self-management information and suggestions (I) +/ Content format (I) +/ User-friendly message tone (I) 🡪 Perceived ease of use (M) - **H3, H4:** T1D self-management information and suggestions (I) 🡪 Knowledge and skills (M) 🡪 Outcome expectancies (M) + Self-efficacy (M) 🡪 Negative emotions (M) 🡪 Transition readiness (O) - **H5:** Transition support information (I) 🡪 Knowledge and skills (M) 🡪 Outcome expectancies (M) 🡪 Negative emotions (M) 🡪 Transition readiness (O) - **H10:** Transition reminders (I) 🡪 Behavioural cueing (M) - **New:** T1D self-management information and suggestions (I) 🡪 Reinforcement (M) 🡪 Behavioural cueing (M) 🡪 Transition readiness (O) |
| ID03 | - Long duration of diabetes - Concerned about not knowing - With intense competing priorities | - **H1:** T1D self-management information and suggestions (I) +/ Transition reminders (I) +/ Transition support information (I) +/ Stress management strategies (I) +/ Personalization (I) 🡪 Perceived usefulness (M) - **H2:** User-friendly message tone (I) 🡪 Perceived ease of use (M) - **H3:** T1D self-management information and suggestions (I) 🡪 Knowledge and skills (M) 🡪 Self-efficacy (M) - **H5:** Transition support information (I) 🡪 Knowledge and skills (M) 🡪 Outcome expectancies (M) 🡪 Behavioural cueing (M) + Self-efficacy (M) 🡪 Negative emotions (M) 🡪 Transition readiness (O) - **H8:** Stress management strategies (I) 🡪 Reinforcement (M) 🡪 Negative emotions (M) 🡪 Blood glucose self-monitoring behaviours (O) - **H10:** Competing priorities (C) + Transition reminders (I) 🡪 Behavioural cueing (M) - **New:** T1D self-management information and suggestions (I) 🡪 Reinforcement (M) |
| ID04 | - Long duration of diabetes - Concerned about not knowing - With intense competing priorities | - **H1:** T1D self-management information and suggestions (I) +/ Transition reminders (I) +/ Transition support information (I) +/ Content format (I) +/ User-friendly message tone (I) +/ Credible sources (I) 🡪 Perceived usefulness (M) - **H2:** T1D self-management information and suggestions (I) +/ Content format (I) +/ User-friendly message tone (I) 🡪 Perceived ease of use (M) - **H3, H4:** T1D self-management information and suggestions (I) 🡪 Knowledge and skills (M) 🡪 Outcome expectancies (M) 🡪 Behavioural cueing (M) + Self-efficacy (M) 🡪 Negative emotions (M) 🡪 Transition readiness (O) - **H5:** Transition support information (I) 🡪 Knowledge and skills (M) 🡪 Reinforcement (M) + Outcome expectancies (M) 🡪 Negative emotions (M) 🡪 Transition readiness (O) - **H10:** Competing priorities (C) + Transition reminders (I) 🡪 Behavioural cueing (M) + Self-efficacy (M) 🡪 Transition readiness (O) - **New:** T1D self-management information and suggestions (I) 🡪 Reinforcement (M) 🡪 Behavioural cueing (M) 🡪 Blood glucose (O) |
| ID05 |  | - **H1:** T1D self-management information and suggestions (I) +/ Personalization (I) +/ Content format (I) +/ Credible sources (I) 🡪 Perceived usefulness (M) - **H2:** T1D self-management information and suggestions (I) +/ Content format (I) 🡪 Perceived ease of use (M) - **H3:** T1D self-management information and suggestions (I) 🡪 Knowledge and skills (M) 🡪 Self-efficacy (M) 🡪 Transition readiness (O) - **H5:** Transition support information (I) 🡪 Knowledge and skills (M) 🡪 Outcome expectancies (M) + Self-efficacy (M) 🡪 Transition readiness (O) - **New:** T1D self-management information and suggestions (I) 🡪 Reinforcement (M) 🡪 Self-efficacy (M) + Outcome expectancies (M) 🡪 Negative emotions (M) 🡪 Behavioural cueing (M) 🡪 Blood glucose self-monitoring behaviours (O) |
| ID06 | - Perceived strong need for knowledge | - **H1:** T1D self-management information and suggestions (I) +/ Transition support information (I) +/ Personalization (I) +/ Content format (I) 🡪 Perceived usefulness (M) - **H2:** T1D self-management information and suggestions (I) +/ Content format (I) +/ User-friendly message tone (I) 🡪 Perceived ease of use (M) - **H3, H4:** T1D self-management information and suggestions (I) 🡪 Knowledge and skills (M) 🡪 Outcome expectancies (M) / Behavioural cueing (M) / Self-efficacy (M) / Negative emotions (M) 🡪 Blood glucose (O) - **H5:** Transition support information (I) 🡪 Knowledge and skills (M) +/ Reinforcement (M) 🡪 Behavioural cueing (M) 🡪 Transition readiness (O) - **New:** T1D self-management information and suggestions (I) 🡪 Reinforcement (M) |
| ID07 | - Long duration of diabetes | - **H1:** T1D self-management information and suggestions (I) +/ Problem-solving support (I) +/ Personalization (I) +/ Content format (I) +/ Credible sources (I) +/ Real-time interactivity (I) 🡪 Perceived usefulness (M) - **H2:** T1D self-management information and suggestions (I) +/ User-friendly message tone (I) 🡪 Perceived ease of use (M) - **H3:** T1D self-management information and suggestions (I) 🡪 Knowledge and skills (M) 🡪 Self-efficacy (M) 🡪 Negative emotions (M) - **H6:** Problem-solving support (I) + Personalization (I) + Credible sources (I) + Real-time interactivity (I) 🡪 Knowledge and skills (M) - **H8:** No significant baseline diabetes distress (C) + Stress management strategies (I) 🡪 No perceived change in negative emotion (M) |
| ID08 | - Short duration of diabetes - Concerned about not knowing | - **H1:** T1D self-management information and suggestions (I) +/ Transition reminders (I) +/ Transition support information (I) +/ Stress management strategies (I) +/ Personalization (I) +/ Content format (I) +/ Credible sources (I) 🡪 Perceived usefulness (M) - **H2:** T1D self-management information and suggestions (I) +/ Personalization (I) +/ User-friendly message tone (I) 🡪 Perceived ease of use (M) - **H3:** T1D self-management information and suggestions (I) 🡪 Knowledge and skills (M) 🡪 Self-efficacy (M) 🡪 Negative emotions (M) - **H5:** Transition support information (I) 🡪 Knowledge and skills (M) 🡪 Outcome expectancies (M) + Self-efficacy (M) 🡪 Negative emotions (M) 🡪 Transition readiness (O) - **H8:** Stress management strategies (I) 🡪 Negative emotions (M) + Reinforcement (M) - **H10:** Transition reminders (I) 🡪 Behavioural cueing (M) 🡪 Transition readiness (O) - **New:** T1D self-management information and suggestions (I) 🡪 Reinforcement (M) 🡪 Self-efficacy (M) |
| ID09 | - Long duration of diabetes - With intense competing priorities | - **H1:** T1D self-management information and suggestions (I) +/ Transition reminders (I) 🡪 Perceived usefulness (M) - **H2:** T1D self-management information and suggestions (I) +/ User-friendly message tone (I) 🡪 Perceived ease of use (M) - **H3:** T1D self-management information and suggestions (I) 🡪 Knowledge and skills (M) 🡪 Behavioural cueing (M) 🡪 Blood glucose self-monitoring behaviours (O) - **H10:** Competing priorities (C) + Transition reminders (I) 🡪 Behavioural cueing (M) 🡪 Transition readiness (O) - **New:** T1D self-management information and suggestions (I) 🡪 Reinforcement (M) 🡪 Behavioural cueing (M) 🡪 Blood glucose self-monitoring behaviours (O) 🡪 Blood glucose (O) - **New:** T1D self-management information and suggestions (I) 🡪 Negative emotions (M) - **New:** T1D self-management information and suggestions (I) 🡪 Behavioural cueing (M) |
| ID10 | - Long duration of diabetes - With intense competing priorities | - **H1:** T1D self-management information and suggestions (I) +/ Transition reminders (I) +/ Personalization (I) +/ Content format (I) +/ User-friendly message tone (I) +/ Credible sources (I) +/ Real-time interactivity (I) 🡪 Perceived usefulness (M) - **H2:** Personalization (I) +/ Content format (I) +/ Real-time interactivity (I) 🡪 Perceived ease of use (M) - **H3, H4:** T1D self-management information and suggestions (I) 🡪 Knowledge and skills (M) 🡪 Outcome expectancies (M) / Behavioural cueing (M) 🡪 Blood glucose self-monitoring behaviours (O) / Transition readiness (O) - **H8:** Positive baseline self-efficacy (C) + No significant baseline diabetes distress (C) + Stress management strategies (I) 🡪 No perceived change in negative emotion (M) - **H10:** Competing priorities (C) + Transition reminders (I) 🡪 Behavioural cueing (M) - **New:** T1D self-management information and suggestions (I) 🡪 Reinforcement (M) 🡪 Behavioural cueing (M) 🡪 Blood glucose self-monitoring behaviours (O) - **New:** T1D self-management information and suggestions (I) 🡪 Negative emotions (M) |
| ID11 |  | - **H1:** T1D self-management information and suggestions (I) +/ Stress management strategies (I) +/ Personalization (I) +/ Content format (I) 🡪 Perceived usefulness (M) - **H2:** T1D self-management information and suggestions (I) +/ Personalization (I) +/ User-friendly message tone (I) 🡪 Perceived ease of use (M) - **H3:** T1D self-management information and suggestions (I) 🡪 Knowledge and skills (M) - **H8:** Stress management strategies (I) 🡪 Negative emotions (M) 🡪 Blood glucose self-monitoring behaviours (O) + Transition readiness (O) - **New:** T1D self-management information and suggestions (I) 🡪 Reinforcement (M) 🡪 Behavioural cueing (M) |
| ID12 | - Long duration of diabetes | - **H1:** T1D self-management information and suggestions (I) +/ Transition reminders (I) +/ Transition support information (I) +/ Stress management strategies (I) +/ Personalization (I) +/ Content format (I) +/ User-friendly message tone (I) +/ Credible sources (I) 🡪 Perceived usefulness (M) - **H2:** T1D self-management information and suggestions (I) +/ Content format (I) +/ User-friendly message tone (I) 🡪 Perceived ease of use (M) - **H3:** T1D self-management information and suggestions (I) 🡪 Knowledge and skills (M) 🡪 Blood glucose self-monitoring behaviours (O) - **H8:** Stress management strategies (I) 🡪 Negative emotions (M) - **H10:** Transition reminders (I) 🡪 Behavioural cueing (M) 🡪 Transition readiness (O) - **New:** T1D self-management information and suggestions (I) 🡪 Reinforcement (M) - **New:** Transition support information (I) 🡪 Reinforcement (M) |
| ID13 | - Long duration of diabetes - Perceived strong need for knowledge | - **H1:** T1D self-management information and suggestions (I) +/ Personalization (I) +/ Content format (I) +/ Credible sources (I) 🡪 Perceived usefulness (M) - **H2:** T1D self-management information and suggestions (I) +/ Personalization (I) +/ Content format (I) 🡪 Perceived ease of use (M) - **H3, H4:** T1D self-management information and suggestions (I) 🡪 Knowledge and skills (M) 🡪 Outcome expectancies (M) / Behavioural cueing (M) / Self-efficacy (M) 🡪 Transition readiness (O) - **New:** T1D self-management information and suggestions (I) 🡪 Reinforcement (M) 🡪 Behavioural cueing (M) 🡪 Blood glucose self-monitoring behaviours (O) |
| ID14 | - With intense competing priorities | - **H1:** T1D self-management information and suggestions (I) +/ Transition reminders (I) +/ Credible sources (I) 🡪 Perceived usefulness (M) - **H2:** T1D self-management information and suggestions (I) +/ Content format (I) +/ User-friendly message tone (I) 🡪 Perceived ease of use (M) - **H3:** T1D self-management information and suggestions (I) 🡪 Knowledge and skills (M) 🡪 Self-efficacy (M) + Behavioural cueing (M) 🡪 Blood glucose self-monitoring behaviours (O) - **H10:** Competing priorities (C) + Memory ability (C) + Transition reminders (I) 🡪 Behavioural cueing (M) 🡪 Transition readiness (O) |
| ID15 | - Long duration of diabetes - With intense competing priorities | - **H1:** T1D self-management information and suggestions (I) +/ Transition reminders (I) +/ Stress management strategies (I) +/ Personalization (I) +/ Content format (I) +/ Credible sources(I) 🡪 Perceived usefulness (M) - **H2:** T1D self-management information and suggestions (I) +/ Personalization (I) +/ Content format (I) +/ User-friendly message tone (I) 🡪 Perceived ease of use (M) - **H3:** T1D self-management information and suggestions (I) 🡪 Knowledge and skills (M) 🡪 Self-efficacy (M) + Negative emotions (M) 🡪 Blood glucose (O)🡪 Transition readiness (O) - **H8:** Stress management strategies (I) 🡪 Negative emotions (M) 🡪 Transition readiness (O) - **H10:** Competing priorities (C) + Transition reminders (I) 🡪 Behavioural cueing (M) 🡪 Transition readiness (O) - **New:** T1D self-management information and suggestions (I) 🡪 Reinforcement (M) 🡪 Behavioural cueing (M) 🡪 Transition readiness (O) |
| ID16 | - Long duration of diabetes - Concerned about not knowing | - **H1:** T1D self-management information and suggestions (I) 🡪 Perceived usefulness (M) - **H2:** T1D self-management information and suggestions (I) +/ Content format (I) 🡪 Perceived ease of use (M) - **H3:** T1D self-management information and suggestions (I) 🡪 Knowledge and skills (M) |
